# Screening of cell-type-specific meta-programs for drug repurposing in Alzheimer’s disease

**DOI:** 10.1093/bib/bbag411

**Published:** 2026-07-27

**Authors:** Chunlong Zhang, Yuxi Zhang, Zhiyi Wu, Yuting Zhang, Fei Xue, Qinglong Tan, Xiaoling Zhong, Yu Zhang, Ziyan Zhao, Yunyi Peng, Hongping Chen, Feng Li, Yunpeng Zhang

**Affiliations:** College of Bioinformatics Science and Technology, Harbin Medical University, No. 157 Baojian Road, Nangang District, Harbin 150081, China; College of Bioinformatics Science and Technology, Harbin Medical University, No. 157 Baojian Road, Nangang District, Harbin 150081, China; College of Bioinformatics Science and Technology, Harbin Medical University, No. 157 Baojian Road, Nangang District, Harbin 150081, China; College of Bioinformatics Science and Technology, Harbin Medical University, No. 157 Baojian Road, Nangang District, Harbin 150081, China; College of Bioinformatics Science and Technology, Harbin Medical University, No. 157 Baojian Road, Nangang District, Harbin 150081, China; College of Bioinformatics Science and Technology, Harbin Medical University, No. 157 Baojian Road, Nangang District, Harbin 150081, China; College of Bioinformatics Science and Technology, Harbin Medical University, No. 157 Baojian Road, Nangang District, Harbin 150081, China; College of Bioinformatics Science and Technology, Harbin Medical University, No. 157 Baojian Road, Nangang District, Harbin 150081, China; College of Bioinformatics Science and Technology, Harbin Medical University, No. 157 Baojian Road, Nangang District, Harbin 150081, China; College of Bioinformatics Science and Technology, Harbin Medical University, No. 157 Baojian Road, Nangang District, Harbin 150081, China; Department of Neurology, The First Affiliated Hospital of Harbin Medical University, No. 23 Youzheng Street, Nangang District, Harbin 150081, China; College of Bioinformatics Science and Technology, Harbin Medical University, No. 157 Baojian Road, Nangang District, Harbin 150081, China; College of Bioinformatics Science and Technology, Harbin Medical University, No. 157 Baojian Road, Nangang District, Harbin 150081, China

**Keywords:** Alzheimer’s disease, meta-program, single cell/nucleus RNA sequencing, multi-omics

## Abstract

Alzheimer’s disease (AD) is a progressive neurodegenerative disorder driven by complex cellular changes. To identify transcriptional signatures involved in AD pathology, we first analyzed nine single-cell/nucleus RNA-seq datasets from 67 high-pathology cases (Braak stages V/VI), generating an atlas of 363 243 cells. Using a multi-sample integration strategy, we identified 51 cross-sample meta-programs (MPs) across seven cell types that collectively capture key AD-related processes, including synaptic dysfunction and neuroinflammation. By screening bulk transcriptomes from 1208 ad and 725 normal samples, we found that 26 MPs (13 pathogenic and 13 protective) displayed differential activity in AD, characterized by up-regulation of microglia MPs and down-regulated of neuron MPs. Furthermore, spatial transcriptomics revealed that these MPs form spatially coherent communities associated with distinct cellular neighborhoods. Finally, we performed an integrative drug repurposing screen to identify candidate drugs predicted to regulate pathogenic MPs. In conclusion, we conducted an integrated multi-omics study to identify AD cell-type-specific MPs, and this framework can be applied to deconvolve cellular heterogeneity and screen candidate drugs for AD.

## Introduction

Alzheimer’s disease (AD) is a devastating neurodegenerative disorder characterized by mild memory impairment to severe cognitive decline [1]. This progression is underpinned by profound alterations in brain cell composition and function. Single-cell and single-nucleus RNA sequencing (sc/snRNA-seq) have revolutionized the profiling of these changes, revealing an unprecedented level of cellular heterogeneity in AD [2–4]. The rapid accumulation of public AD sc/snRNA-seq datasets enables the systematic, cross-study exploration of disease-associated transcriptional states.

Non-negative matrix factorization (NMF) has proven to be a powerful tool for identifying interpretable, biologically relevant gene expression programs. Its application in AD research has been diverse, ranging from diagnostic image analysis [5], to disease subtyping [6]. The versatility of NMF is further underscored by its adoption in various genomic applications. In oncology, NMF has been utilized in dissecting cellular heterogeneity. Kinker *et al.* applied NMF to multiplexed single-cell data from ~200 cell lines, revealing 12 conserved expression programs that underlie recurrent heterogeneity across diverse cancer types [7]. Scaling this approach further, Gavish *et al.* integrated data from 77 studies across 1163 tumor samples and 24 cancer types to define 41 consensus meta-programs (MPs) within malignant cells using a single-sample NMF strategy [8]. Despite the extensive and successful application of NMF-based strategies to characterize cell-type-specific transcriptional programs in cancer, similar systematic efforts to analyze the complex cellular landscape of AD at single-cell resolution are notably lacking.

**Figure 1:**
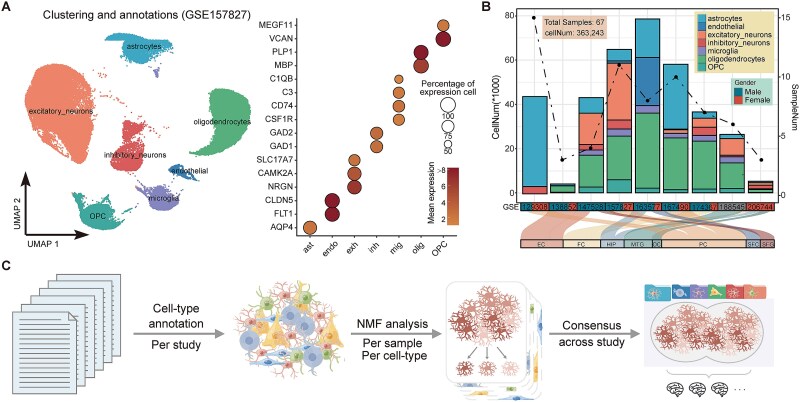
Curated collection of sc/snRNA-seq datasets and framework for MP identification. (A) Cell annotations of the dataset of Lau *et al.* (GSE157827). Left: Uniform Manifold Approximation and Projection (UMAP) plot of all cells, colored by cell type. Right: Dot plots showing expression of canonical marker genes for each cell type. (B) Bar plot showing the cell number per cell type across datasets. The x-axis indicates datasets, with color indicating whether sex information is available (male: blue, female: red). The lower panel of the Sankey plot displayed brain region information for each dataset. (C) Schematic overview of the framework for identifying robust cell-type-specific MP.

To address this gap, we applied a computational strategy to multiple single-cell transcriptomic datasets comprising high-pathology samples with neuropathologically confirmed diagnoses, aiming to define a comprehensive atlas of disease-associated transcriptional programs across all major brain cell types. Firstly, we annotated and integrated 363 243 cells from nine sc/snRNA-seq datasets of 67 high-pathology (Braak V/VI) samples. Using a single-sample NMF strategy, we identified 51 cell-type-specific transcriptional MPs as the foundational transcriptional units. Building on these 51 MPs, we integrated bulk transcriptome datasets to assess their disease relevance, which allowed us to distinguish pathogenic MPs and protective MPs. Next, we leveraged spatial transcriptomics to characterize the spatial organization of these MPs and to delineate their spatial relationships with cell types. Finally, focusing specifically on the 13 pathogenic MPs, we applied an integrative strategy to nominate candidate drugs for AD. Our work provides a comprehensive framework and resource for deciphering the fundamental transcriptional units and multicellular interplay underlying AD pathophysiology.

## Results

### Single-cell atlas of AD high-pathology samples

Firstly, we aggregated single-cell/nucleus transcriptome data from nine publicly available studies. Specifically, to capture transcriptional signatures aligned with AD pathology, we further focused exclusively on 67 high-pathology AD samples from donors with Braak stages V and VI (see *Materials and Methods*, Table S1). Stringent quality control was applied to filter out low-quality cells, doublets, and ambiguous clusters, after which cells were annotated using established marker genes. As shown in Fig. 1A (using GSE157827 as an example) and across all datasets in Supplementary Fig. S1, we successfully annotated seven major brain cell types, including astrocytes, endothelial cells, excitatory neurons, inhibitory neurons, microglia, oligodendrocytes and oligodendrocyte precursor cells (OPCs). Our final integrated dataset comprised 363 243 high-confidence cells; the distribution of these cells across datasets, brain regions, and sexes is detailed in Fig. 1B.

### Identification of cross-sample transcriptional meta-programs

**Figure 2:**
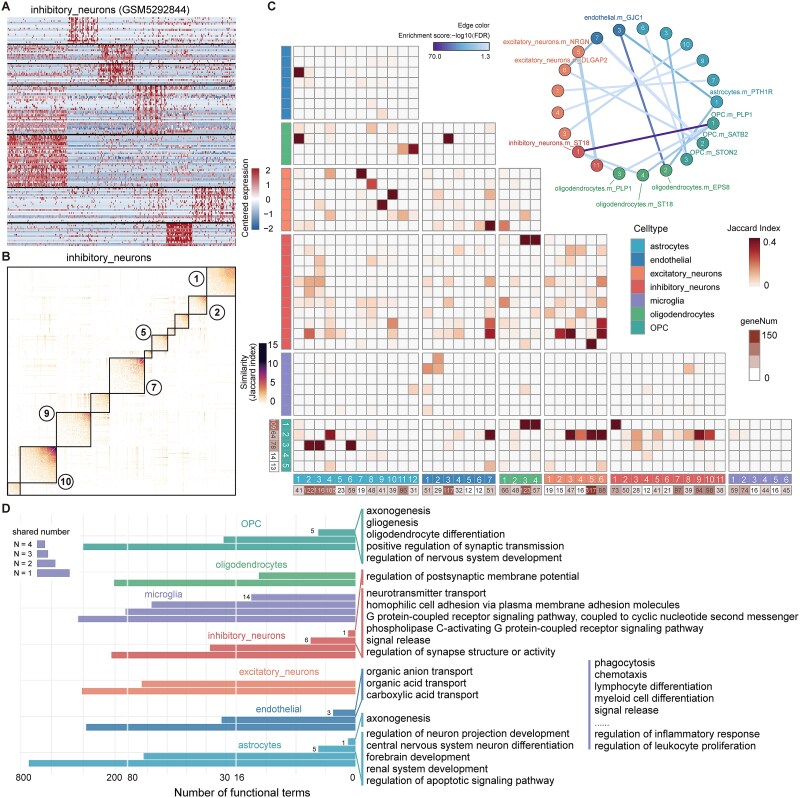
Cell-type-specific MPs and their functional annotations. (A) Heatmap showing expression levels in inhibitory neurons of representative sample (GSM5292844). Genes are grouped by module membership, with horizontal lines delineating module boundaries. (B) Heatmap showing the significance of the overlap between individual modules derived from inhibitory neurons, as measured by Jaccard similarity indices. (C) Similarity heatmap showing the Jaccard similarity indices between MPs. MP labels denote the MP number and the number of genes within each MP. The upper-right corner displayed a network of MPs with significantly overlapping gene sets (hypergeometric test). (D) Bar plot of shared functional terms across multiple MPs per cell type. The bar height indicates the count of overlapping terms, with representative terms listed on the right.

First, to capture cell-type-specific transcriptional programs, we analyzed each major cell type independently. For each cell type, we utilized a multi-sample integration strategy to identify robust transcriptional characterizations that are reproducible across samples (see *Materials and Methods*, Fig. 1C). This approach avoids the technical pitfalls of direct dataset merging, instead, we first analyzed each sample individually by applying NMF to every AD sample to identify sets of co-varying genes, termed ‘modules’ (e.g. six modules identified in a sample for inhibitory neuron, Fig. 2A). For robustness of following results, the samples with less than 50 cells were removed and the final sample number information for each cell type was provided in Supplementary Table S2. Next, we performed a comparative analysis of gene composition across all sample-derived modules. This allows us to define MPs as gene sets that exhibit recurrent co-expression patterns across multiple samples (e.g. inhibitory neuron MPs shown in Fig. 2B).

Applying this strategy across all seven cell types, we identified a total of 51 MPs (astrocytes: 12; endothelial cells: 7; excitatory neurons: 6; inhibitory neurons: 11; microglia: 6; oligodendrocytes: 4; OPCs: 5), ranging in size from 10 to 317 genes (Fig. 2C, Supplementary Fig. S2 and Supplementary Table S3). To facilitate interpretation, we adopted a systematic naming convention: each MP was designated by its cell type of origin and the most frequently occurring gene among its constituent modules (e.g. the inhibitory neuron MP inNeu.m_FGF13). To show the robustness of our approach and ensure that these MPs represent biological signals rather than technical artifacts, we assessed their cross-dataset reproducibility. As a representative example, we projected all inhibitory neurons from six independent datasets into a shared low-dimensional space and visualized the activity of the identified MPs (Supplementary Fig. S3). The majority, including inNeu.m_FGF13 and inNeu.m_GRM1, exhibited activity patterns across multiple datasets. This concordance confirms that our multi-sample integration strategy effectively disentangles dataset-specific technical noise from biologically recurrent transcriptional states.

To assess the cell-type specificity of the identified MPs, we quantified the degree of gene overlap between MPs derived from different cell types. As shown in Fig. 2C, only 16 significant cross cell-type gene relationships were detected among all MP pairs, indicating that the vast majority of MPs exhibit cell type specificity. Notably, microglia MPs showed no significant overlap with MPs from any other cell type, supporting their unique transcriptional identity. Furthermore, we compared our MPs with previously published microglia gene signatures from multiple studies and found that our MPs shared gene components with these established features (Supplementary Fig. S4). Gene Ontology (GO) enrichment analysis revealed that these MPs are enriched for fundamental and cell-type-specific biological processes implicated in AD pathophysiology (Fig. 2D and Supplementary Table S4). Specifically, most MPs exhibited distinct biological functions; however, within the same cell type, multiple MPs also shared certain functional features. For example, axonogenesis, gliogenesis and oligodendrocyte differentiation were identified in three OPC MPs. Similarly, astrocyte MPs shared functions such as axonogenesis and some functions about neuron projection and differentiation. Microglial MPs were more enriched for phagocytosis, chemotaxis, and regulation of inflammatory response. These glial MPs were involved in neuroinflammation and axonogenesis, underscoring the regulatory role of glial cells in AD neuroimmunology. Inhibitory neuron MPs showed enrichment for neurotransmitter transport and regulation of synapse structure or activity. These results highlight both the specificity and the shared functional architecture across MPs within and between cell types.

### Screening of disease-associated MPs based on bulk transcriptome data

It is important to note that despite the use of high-grade AD samples (Braak stage V-VI), the cell-type-specific MPs we identified harbor transcriptional signatures from both normal and disease states. To investigate their disease relevance between AD and normal conditions, we obtained 10 bulk transcriptome datasets [9–14] comprising 1208 ad and 725 normal samples in total (Supplementary Table S5). For each dataset, two types of analyses were conducted: MP activity analysis and gene overlap analysis. In the MP activity analysis, we first calculated the activity score of each MP using ssGSEA, and then applied the limma [15] to assess the differential activity of each MP between AD and normal conditions. In the gene overlap analysis, we similarly used limma to identify differentially expressed genes (DEGs) between AD and normal conditions in each dataset, and then performed hypergeometric tests to evaluate the significance of overlap between the DEGs and the gene sets of each MP. In both differential analyses, we adjusted for the available confounders within each dataset, including age, sex, and brain region.

**Figure 3:**
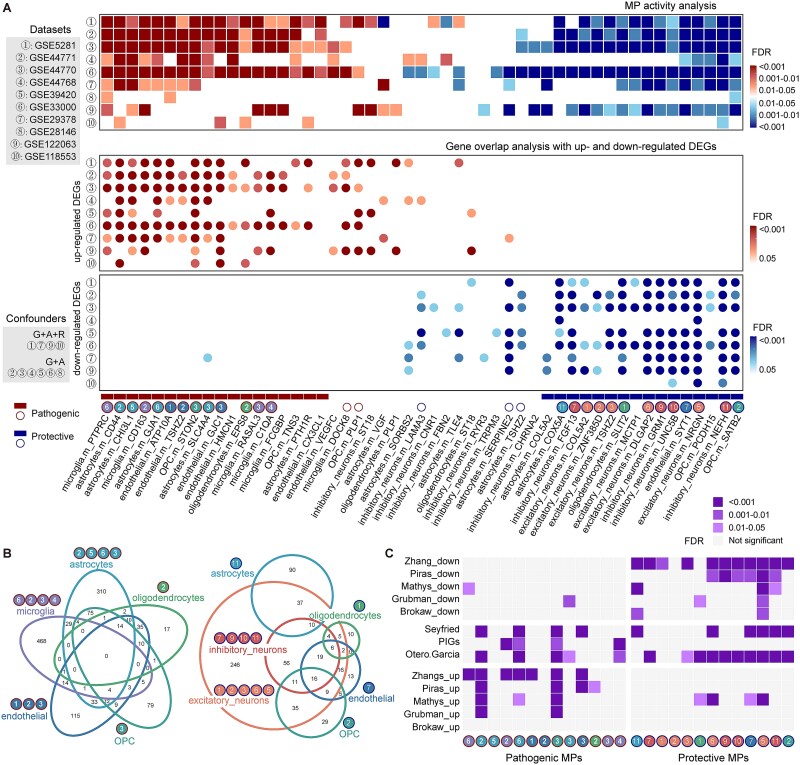
Bulk transcriptome data defines AD-related MPs. (A) Two vertically heatmaps are shown for each dataset. The upper heatmap displays MP activity difference: each row represents an MP, each column a dataset, and colors indicate the significance of differential activity calculated by limma (red: up-regulated, blue: down-regulated). The lower heatmap shows gene overlap significance: each row represents an MP, each column a dataset, and colors indicate the significance of gene set overlap calculated by the hypergeometric test. Dataset IDs are indicated in the upper-left corner, and confounder information (G: gender, A: age, R: brain region) for each dataset is provided in the lower-left corner. (B) Venn diagram showing the overlap of significant functional terms associated with pathogenic MPs and protective MPs. (C) Heatmap showing the overlap between pathogenic, protective MPs and previously published AD-related gene signatures. Colors represent the significance of overlap calculated by the hypergeometric test.

As shown in Fig. 3A, 26 out of 51 MPs exhibited significant differential activity in at least four datasets from both above analyses. Specifically, 13 MPs showed both higher activity scores in AD and significant overlapping with up-regulated DEGs (defined as pathogenic MPs), while 13 showed higher scores in normal samples and significant overlapping with down-regulated DESs (defined as protective MPs). Among these significant MPs, all excitatory and inhibitory neuron MPs were protective, suggesting a loss of their associated functions during AD onset. Furthermore, four microglia MPs were all pathogenic, showing the neuroinflammation is a potential AD driver. MPs from astrocytes, oligodendrocytes, OPC and endothelial cells exhibited two distinct disease-associated patterns. The functional relationships among pathogenic and protective MPs were also explored (Fig. 3B). By assessing the overlap between our disease-associated MPs and published AD-related signatures, we also observed consistent results, including the direction of dys-regulation, namely, pathogenic MPs were up-regulated, while protective MPs were down-regulated (Fig. 3C). Furthermore, we evaluated the associations with key neuropathological traits in a well-phenotyped bulk transcriptomic dataset (GSE84422) [16] with available measures of amyloid load, tau tangle density, and cognitive decline. As shown in Supplementary Fig. S5, most pathogenic MPs displayed positive associations with neurofibrillary tangles density and clinical dementia score. Notably, although belonging to the pathogenic ones, astro.m_CH3L1 and endo.m_ATP10A displayed negatively associated early progression features, including neuritic plaque density and cerad rating scores. It was shown that these two MPs might not be involved in the early stage of AD onset. And the negative associations were also observed between protective MPs and these features.

### Spatial transcriptomics reveals cellular organization heterogeneity in AD

**Figure 4:**
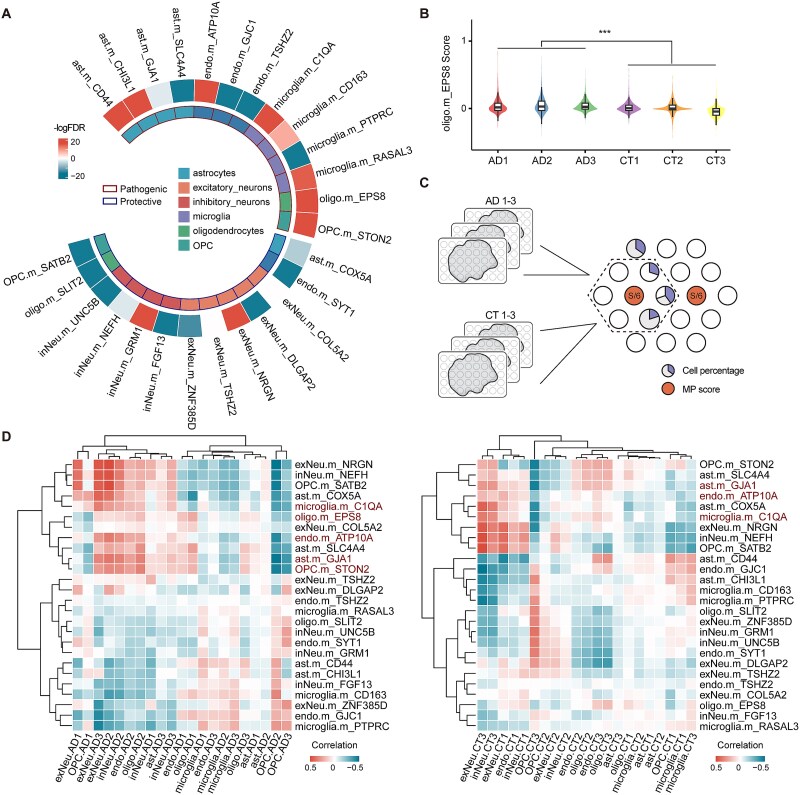
Spatial transcriptome analysis of disease-related MPs. (A) Circos plot showing differential MPs between AD and control in spatial transcriptomics data, with red indicating AD-upregulated MPs and blue indicating control-enriched MPs. Inner sectors represent corresponding cell types. (B) Violin plot shows oligo.m_EPS8 activity score across AD and normal samples. (C) Schematic where orange spots indicate MP activity, and gray spots (within dashed circles around the orange spot) indicates the percentage of cells of interest. (D) Heatmap showing the correlations between MP activity and the abundance of neighboring cell types across AD (left) and control (right) samples. Colors indicate spearman correlation coefficients, with red denoting positive associations and blue denoting negative associations.

To investigate the spatial organization and cellular crosstalk of these disease transcriptional MPs, we analyzed human spatial transcriptomics data which contained three AD samples and three normal controls. We found that pathogenic associated MPs were generally highly expressed in AD samples, whereas protective associated MPs were highly expressed in control samples (Fig. 4A), as exemplified by olig.m_EPS8 (Fig. 4B). Next, we applied SPOTlight [17] to deconvolve the spatial transcriptomics spots using a reference scRNA-seq dataset (GSE157827), thereby estimating the abundance of seven major cell types per spot. A neighborhood score was calculated for each cell type, representing its local abundance surround a given spot (Fig. 4C). By correlating MP activity scores with these neighborhood profiles across all spots, we mapped how specific transcriptional states correspond to distinct cellular microenvironment between AD and normal conditions. As shown in Fig. 4D, several pathogenic MPs including microglia.m_C1QA, oligo.m_EPS8, endo.m_ATP10A, ast.m_GJA1, and OPC.m_STON2 showed a positive spatial correlation with excitatory neurons and inhibitory neurons in AD condition. However, in normal brain tissue, ast.m_GJA1, endo.m_ATP10A, and microglia.m_C1QA showed little or no significant spatial correlation with these cell types. These findings suggest the formation of disease associated multicellular niches, where activated transcriptomic MPs from glial and endothelial cells engage in extensive interactions with multiple neuronal populations. This spatial concordance suggests that these MPs may collectively represent a pathologically coordinated niche, potentially driven by shared micro-environmental cues as a novel neurodegenerative hallmark.

### Candidate drug prioritization based on AD-associated MPs

**Figure 5:**
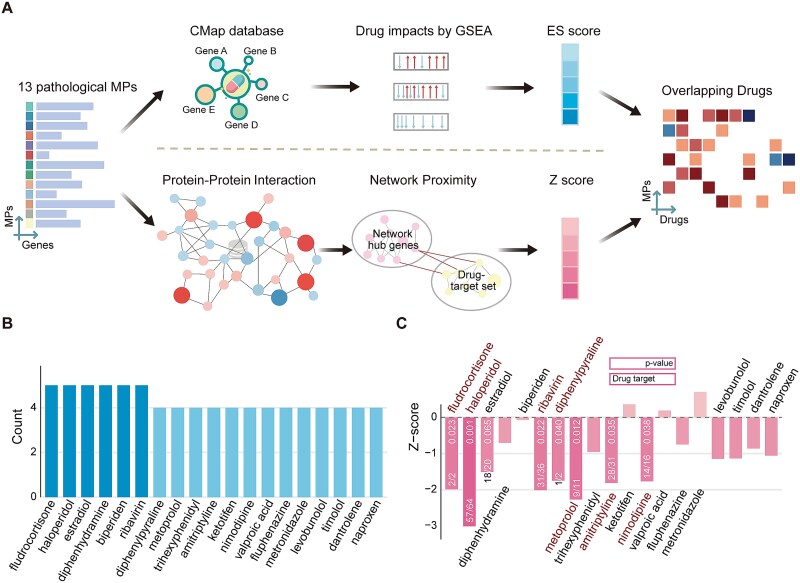
Drug repurposing based on AD pathogenic MPs. (A) Two in silico perturbation methods for drug repurposing. GSEA leverages drug–gene signatures from the CMap database and DEGs to calculate the ES. The ES reflects the drug’s potential to reverse the observed gene expression patterns. Network proximity analysis identifies potential drugs by assessing the proximity between the drug target set and hub gene set. Drug efficacy is evaluated based on the proximity distance and Z-score. (B) The candidate drug with significant GSEA results from at least four cell types. (C) The network proximity results of 19 candidate drugs identified in B. The drug target number and significance was displayed for some representative drugs. ES, enrichment score.

To systematically identify candidate drugs capable of targeting AD-associated MPs, we integrated two orthogonal computational strategies: Connectivity Map (CMap)-based transcriptional perturbation analysis and network proximity analysis (see *Materials and Methods*, Fig. 5A). For CMap analysis, we grouped 13 pathogenic into 5 gene sets by cell type, and performed the gene set enrichment analysis (GSEA) to evaluate whether the drug-induced transcriptional perturbation could significantly down-regulate the risk gene set at the level of cell types. For network analysis, we firstly constructed a sub-network based on a high-quality protein–protein interaction (PPI) network using 13 pathogenic MPs and obtained the hub genes with nodes more than four. And then, we assembled a blood–brain barrier permeable drug-target associations comprising 802 drug molecules from public sources [18]. Finally, we performed network-based drug repurposing by integrating hub genes with drug targets within global PPI network. This approach is predicated on the hypothesis that if the average path length between a drug target and a hub gene is significantly shorter than expected by chance, the drug with lower Z-score is likely to exert direct or indirect effects on AD-relevant MPs.

After CMap screening, we identified 19 candidate drugs that reverse the expression levels of risk genes in at least four of the five cell types with normalized enrichment score (NES) < 0 and FDR < 0.05 (Fig. 5B). Furthermore, we utilized network-based strategy to optimize these 19 drugs by evaluating the network proximity between drug targets and 38 hub genes, which was identified from risk sub-network (see Materials and Methods, Supplementary Table S6). As shown in Fig. 5C, seven drugs displayed significant network proximity with *P*-values <.05. Among the seven significant drugs, ribavirin (Z = −2.01, *P*-value = .022) showed the strongest evidence for AD repurposing. Qiao *et al.* demonstrated that ribavirin effectively inhibited HSV-1 replication and reversed multiscale AD-like neuropathology, including Aβ deposition, reactive gliosis, and neuronal loss, in human iPSC-derived HSV-1-infected brain organoid models [19]. Nimodipine (Z = −1.76, *P*-value = .038), an L-type calcium channel blocker with high lipophilicity and blood–brain barrier permeability, showed cognitive benefits for vascular and mixed dementia [20]. Moreover, some marginally significant drugs also displayed the evidence for AD treatment. For instance, estradiol (Z = −1.51, *P*-value = .066), though not reaching nominal significance, has garnered substantial evidence for its AD neuroprotective effects supported by epidemiological cohorts and human iPSC-derived neural models [21, 22]. Dantrolene (Z = −0.86, *P*-value = .195), a ryanodine receptor antagonist, restored neurogenesis and synaptogenesis in AD patient iPSC-derived neurons and nearly eliminated Morris water maze memory deficits in 3xTg-AD transgenic mice [23, 24]. Collectively, these findings demonstrate the utility of our AD-associated MPs as actionable targets for drug prioritization. The successful identification of ribavirin and estradiol, each supported by clinical evidence for AD, validates the translational value of cell-type-specific MPs in therapeutic development.

## Materials and methods

### Collection and processing of AD sc/snRNA-seq datasets

#### Data collection and rationale

To identify cell-type-specific transcriptional MPs that are robust across studies, we systematically curated sc/snRNA-seq datasets from the Gene Expression Omnibus (GEO) database. We focused on datasets containing samples with high neuropathological burden, specifically Braak stage V and VI, to enrich for disease-relevant signals. This curation yielded nine independent AD datasets (2 scRNA-seq and 7 snRNA-seq studies) [25–33]. The sources, brain regions, and key characteristics of each dataset are summarized in Supplementary Table S1.

#### Quality control and data processing

For each dataset, raw gene expression matrices were downloaded. We retained cells with nCount_RNA > 500 and percent.mt < 0.7%. Cells with high ribosomal read fraction (>30%) were also excluded optionally. The filtered data were then normalized and scaled using the Seurat package [34]. The top 3000 highly variable genes were identified using the vst method for downstream analysis.

#### Cell type annotation and harmonization

Dimensionality reduction was performed using principal component analysis with the first 30 components, followed by cell clustering on a shared k-nearest neighbor graph via the Louvain algorithm. To ensure accurate and high-quality annotation across datasets, we employed a dual-strategy approach:

i) Manual annotation: clusters were manually assigned to one of seven major brain cell types based on the expression of well-established marker genes: Excitatory neurons: NRGN, SLC17A7; Inhibitory neurons: GAD1, GAD2; Oligodendrocytes: MBP, MOBP, PLP1, MOG; OPCs: VCAN, MEGF11, PDGFRA, SOX10; Microglia: CSF1R, CD74, C3, HLA-DRA, CX3CR1, C1QB; Endothelial cells: FLT1, CLDN5; Astrocytes: AQP4, SLC1A2.
ii) Automated validation: we further cross-validated these annotations using cellAssign [35], a probabilistic model that assigns cell types based on the marker gene lists same as manual annotation. Only cells with consistent labels from both methods were retained for subsequent analysis. To further enhance cell quality, we retained only clusters in which ≥60% of cells belonged to the same annotated type, effectively filtering out ambiguous clusters and potential doublets.

### Algorithm for MP identification

Our strategy for identifying MPs was designed to first extract co-expressed gene sets from individual samples prior to cross-study integration. This approach minimizes the impact of technical batches by focusing on intrinsic biological patterns within each sample before data aggregation.

#### Step 1: Sample-specific module detection via NMF

For each cell type in every AD sample, we applied NMF to the preprocessed expression matrix (with negative values set to zero), restricting analysis to samples containing at least 50 cells of that type. The nsNMF algorithm from the NMF package [36] was used for this analysis. To determine the optimal latent dimensionality, we performed factorization across a range of values for rank (from 3 to 15). For each factorization, we derived two gene-ranking lists: one ranking genes by their contribution to each factor, and another by each gene’s contribution to the factors. We iteratively assigned genes to a factor based on their contribution, discarding any factors with fewer than five assigned genes. The final rank (i.e. the number of modules) for each sample-cell type combination was selected as the highest k for which the number of stable modules equaled k.

#### Step 2: Cross-sample integration to define MPs

To focus on reproducible signals, we first filtered the modules, retaining only those that exhibited a minimum Jaccard overlap of 5% with at least two other modules. We then constructed a gene–gene co-occurrence network, where an edge was drawn between two genes if they co-occurred in two or more modules. Genes with fewer than three connections were pruned. The resulting network was partitioned into gene communities using the Infomap clustering algorithm from igraph package. Communities containing fewer than five genes were filtered out and the remaining robust gene clusters were finally defined as MPs. Each MP was named by combining the cell type with the gene that appears most frequently across all contributing modules (e.g. inNeu.m_FGF13).

### Microglia gene-sets and AD-related disease signatures

To perform the robust analysis, we obtained established microglia signatures from a previous study [37]. And the gene overlapping between these signatures and our six microglia MPs were performed using hypergeometric test. Similarly, to perform the AD relevance analysis, we obtained AD-related signatures from another study [38]. Then, we assessed the gene overlap between these signatures and our pathogenic/protective MPs using hypergeometric test.

### Calculation of MP activity scores

#### Single-cell level scoring

For a given MP gene set within a cell, we computed its average centered expression, which was compared against a null distribution generated from 1000 random gene sets of matched average expression level. The *P*-value was defined as the proportion of random sets whose average expression exceeds that of the observed MP set. The final score was computed as -log_10_(*P*-value) and linearly rescaled to a range of 0 to 1.

#### MP assignment in integrated space

To visualize MP activity across datasets, we merged all processed expression matrices for a given cell type. Technical batch effects across studies were harmonized using the Harmony algorithm [39]. For each cell, scores were calculated for all MPs of its corresponding cell type. Each cell was then assigned to the MP with the highest activity score, thereby defining discrete transcriptional state within this cell type.

#### Bulk-cell level scoring

To assess the MP activity differences between AD two conditions and AD clinical relevance based on bulk transcriptome datasets, we utilized the GSVA package [40] to calculate the MP activity score. The gsva function was used to perform enrichment scoring for each MP. The resulting scores reflect the activity level of each MP at the sample level in both AD and normal samples.

### Functional enrichment analysis

For functional characterization, GO enrichment analysis was conducted using the R package clusterProfiler [41]. Enrichment of Biological Process terms was assessed using the enrichGO function with Benjamini–Hochberg multiple-testing correction. Significantly enriched terms were identified using an adjusted *P*-value cutoff of .05. Human gene symbols were used as input, and gene annotations were based on the org.Hs.eg.db database.

### Brain spatial transcriptome analysis

We obtained one human brain spatial transcriptomic data from the GEO database [38]. And then, we utilized the SPOTlight to perform the cell type annotation. Reference data from Jiang *et al.* (GSE157827) was utilized to infer the cellular composition of each spot. In addition, spots were also evaluated for the expression of each MP using the AddModuleScore function in Seurat. Distances between spots were computed based on Euclidean distance on pixel coordinates, with scaling such that one unit represents inter-spot distance (<100 μm). The neighborhood of a spot was defined as all spots within a distance less than 1 unit (excluding the spot itself), resulting in neighborhoods of up to six spots. For each spot, a linear regression model was utilized to detect interactions between MP activity and cell type composition, providing insights into their spatial relationships between AD and normal samples.

### Cmap-based drug repurposing

Drug-perturbation gene expression profiles were obtained from the CMap database [42], and a gene-drug effect-size matrix was constructed. The 13 pathogenic MPs were grouped by their cell type of origin into 5 categories: astrocytes (4 MPs), microglia (4 MPs), endothelial cells (3 MPs), oligodendrocytes (1 MP), and OPCs (1 MP). Genes from MPs within each group were merged to generate five cell-type-specific risk gene sets.

For each drug, GSEA was performed for each of the five gene sets using fgseaMultilevel. For the risk gene sets, the expected enrichment direction was negative (NES < 0), meaning that a drug should down-regulate risk genes. By considering the FDR < 0.05, we then obtained candidate drugs with at least two instance for each cell type. The number of cell types with significant enrichment was then counted, and drugs with *N* ≥ 4 were retained and then selected for following network proximity analysis.

### Network-based drug repurposing

#### Protein–protein interaction network and pathogenic sub-network

The PPI network was obtained from a previous study [43] that integrated 12 common databases, including BioGRID, DFCI_NET_2016, HI-II-network, HPRD, InnateDB, INstruct, IntAct, KinomeNetworkX, MINT, PhosphositePlus, PINA, and SignaLink2.0. To ensure robustness in downstream analyses, only PPI interactions supported by at least two of the source databases were retained, forming the final consensus PPI network. By mapping all genes from 13 pathogenic MPs to the PPI network, we can obtained a sub-network, in which each edge was connected by two pathogenic genes. And the pathogenic hub genes were defined as the genes with node degree more than four.

#### Network proximity

We leveraged the relationships between drugs, their targets, and pathogenic hub genes for drug repurposing. This approach identified potential drug candidates that can be repurposed to treat disease based on their proximity to hub genes within a network graph. We first calculated the degree distribution for both drug targets and hub genes, which represents the number of connections each node has in the network. This ensures that nodes randomly selected for baseline comparison maintains similar connectivity properties. Next, we calculated the proximity between two sets of nodes (drug targets and hub genes) based on their shortest-path distances. We then computed the mean of the minimum distances between each drug target and each hub gene. Given a set of drug targets T and a set of hub genes G, the proximity score dTG is calculated:


$${d}_{TG}=\frac{1}{| T|}\sum_{t\in T}{}^ {min\_d}{}_{\kern-1pc g\in G}\ (t,g)$$

where *d*(*t,g*) denotes the shortest path length between nodes *t* and *g* in the network.

As a baseline for comparison, we generated a null distribution of proximity values by randomly selecting nodes from the network while matching the degree distributions of the actual drug targets and hub genes. We repeated this process 500 times, each time calculating the proximity between the randomly sample node sets. We then computed a Z-score of the observed proximity as:


$$z=\frac{d_{TG}-{\mu}_{rand}}{\sigma_{rand}}$$

indicates that drug targets are closer to the hub genes than expected by chance. *P*-values were obtained from the standard normal distribution based on the Z-scores.

## Discussion

In this study, we constructed a comprehensive map of cell-type-specific transcriptional MPs in the AD brain, which captured fundamental transcriptional states within and across seven major cell types. To gain mechanistic insights from the identified MPs, we pursued three complementary lines of investigation. First, we utilized 10 bulk transcriptome data to analyze the disease relevance of these MPs, and defined 13 pathogenic and 13 protective MPs. And their relationships with AD pathological features were characterized. Second, we employed spatial transcriptome to delineate the disease MPs’ cellular organization between AD and normal conditions. Finally, based on the pathogenic MPs, we utilized a computational strategy which integrated network topology and cMap data to generate a prioritized list of candidate drugs for AD.

To ensure that the transcriptional signatures most faithfully represent established AD pathology, we restricted our analysis to definite, high-pathology samples (Braak stages V/VI). Nevertheless, only 26 of the 51 identified MPs were ultimately screened as disease-relevant. The remaining 25 without different activity in bulk data represent constitutive cellular functions. Another reason might the technical artifacts such as low signal-to-noise ratios in specific dataset. This finding underscores the complexity of cellular dysregulation in AD and necessary of integrating multi-omic data to explore the disease heterogeneity. To strengthen the mechanistic link between MPs and AD lesions, we further interrogated our spatial transcriptomic analyses. The expression patterns of several key AD-associated MPs showed different cell type neighbor between AD and normal samples. Further high-resolution spatial methods (e.g. MERFISH or Xenium) will be required to dissect causal relationships at subcellular resolution.

Despite these advances, several limitations warrant consideration. First, by focusing on high-pathology stages, our study captured only the cellular transcriptional features at late stage. How these MPs emerge and evolve from pre-symptomatic to early-stage remains an important question for further investigations. Second, the NMF employed favors the detection of programs characterized by moderate-to-large gene sets and robust co-expression, potentially overlooking those defined by sparse gene signatures. Complementary use of epigenomic data (e.g. scATAC-seq) wound further help identify low-rank but biologically critical programs. Third, our drug repurposing predictions, while supported by existing clinical data, remain purely computational. In vitro or in vivo validations are indispensable to confirm the functional roles of key MPs and the efficacy of candidate drugs. The current study, nevertheless, offers a systematic insight for AD cell-type specific heterogeneity, and we anticipate that this MP-centric framework will pave the way for a novel understanding of other diseases.

**Key Points**

- Identification of 51 robust meta-programs (MPs) revealing cell-type specific dysregulation in Alzheimer’s disease.
- Bulk transcriptome data revealed 13 MPs were defined as pathogenic and 13 as protective.
- Screening of candidate drug via an integrative computational strategy.

## Supplementary Material

Supplementary_material_bbag411

## Data Availability

AD sc/snRNA-seq, bulk, and spatial transcriptome datasets were all obtained from GEO database. The information of these transcriptome datasets were provided in Supplementary Table. The code for cell-type-specific MP identification and spatial transcriptome analyses have been made available on a GitHub repository (https://github.com/Zhangyx-q/AD_MPcode).
