# Supplementary material for: Screening of cell-type-specific meta-programs for drug repurposing in Alzheimer’s disease
Source: Brief Bioinform. 2026 Jul 27;27(4):bbag411. doi: 10.1093/bib/bbag411 (PMC13403181; doi:10.1093/bib/bbag411)
Supplement: Supplementary_material_bbag411 [file supplementary_material_bbag411.zip › Supplementary_Figure_and_Table_caption_bbag411.docx]

**Figure S1.** Cell annotation UMAP plots of nine sc/snRNA-seq datasets.

**Figure S2.** Gene similarity-based clustering results of seven cell types.

**Figure S3.** UMAP embedding of 15,944 inhibitory neurons from AD samples, colored by different datasets (left) and the highest-scoring MPs (right).

**Figure S4.** The dot plot showing the overlap between six microglia MPs and previously published microglia signatures. Colors represent the significance of overlap calculated by the hypergeometric test.

**Figure S5.** Heatmap showing the correlations between MP activity and the AD pathological features in GSE84422-GPL96 (left), GSE84422-GPL97 (middle) and GSE84422-GPL570 (right). Colors indicate Spearman correlation coefficients, with red denoting positive associations and blue denoting negative associations.

**Table S1.** The detailed samples information of the sc/snRNA-seq data.

**Table S2.** Number of cells per cell type and sample number in each dataset.

**Table S3.** The detailed genes in 51 MPs.

**Table S4.** GO enrichment analysis results for 51 MP genes.

**Table S5.** The information of AD bulk transcriptome data.

**Table S6.** The sub-network of pathogenic genes.
