# Supplementary figures and images for: Screening of cell-type-specific meta-programs for drug repurposing in Alzheimer’s disease
Source: Brief Bioinform. 2026 Jul 27;27(4):bbag411. doi: 10.1093/bib/bbag411 (PMC13403181; doi:10.1093/bib/bbag411)

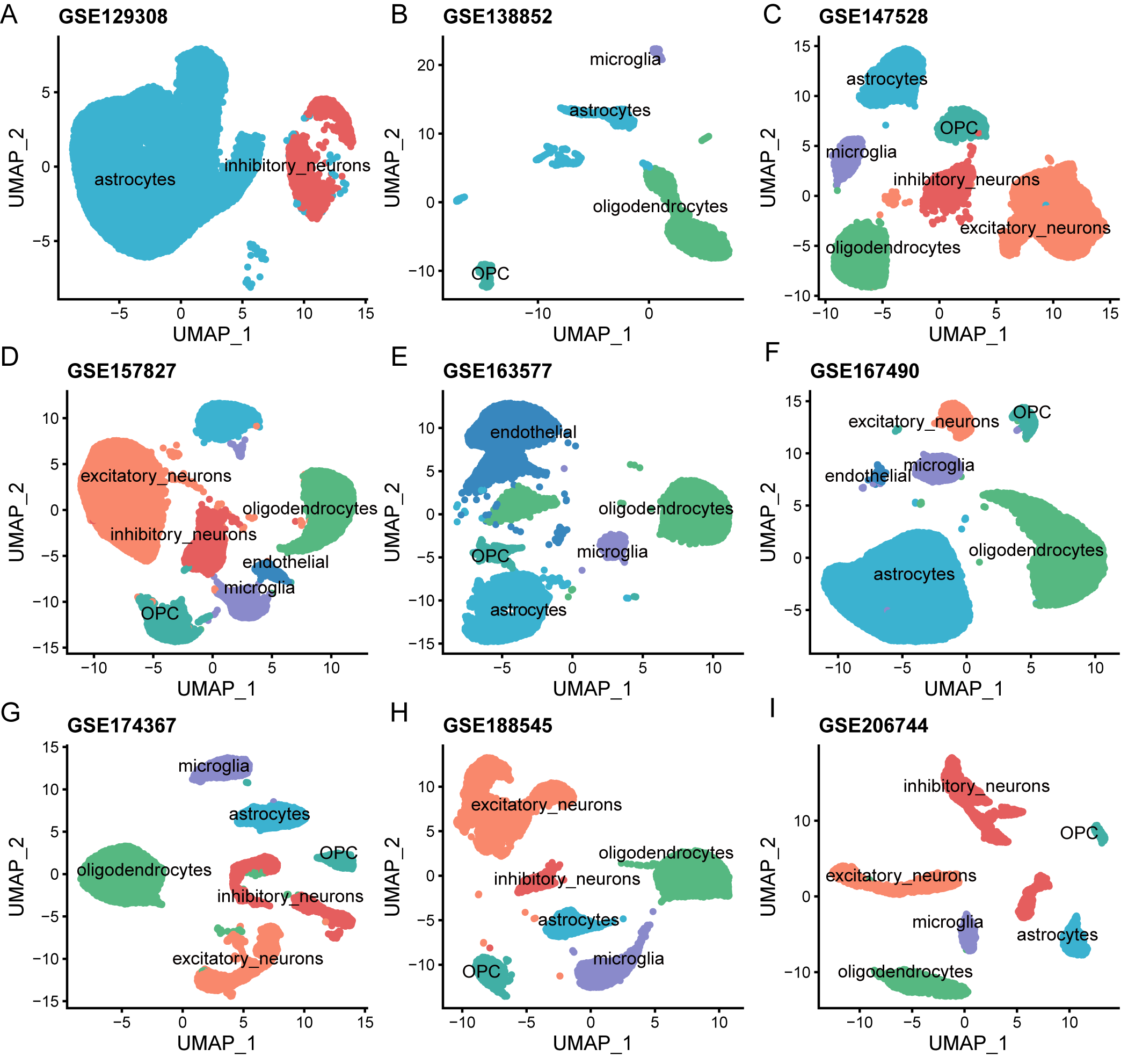

Supplement: Supplementary_material_bbag411 [file supplementary_material_bbag411.zip › Figure_S1_bbag411.tif]

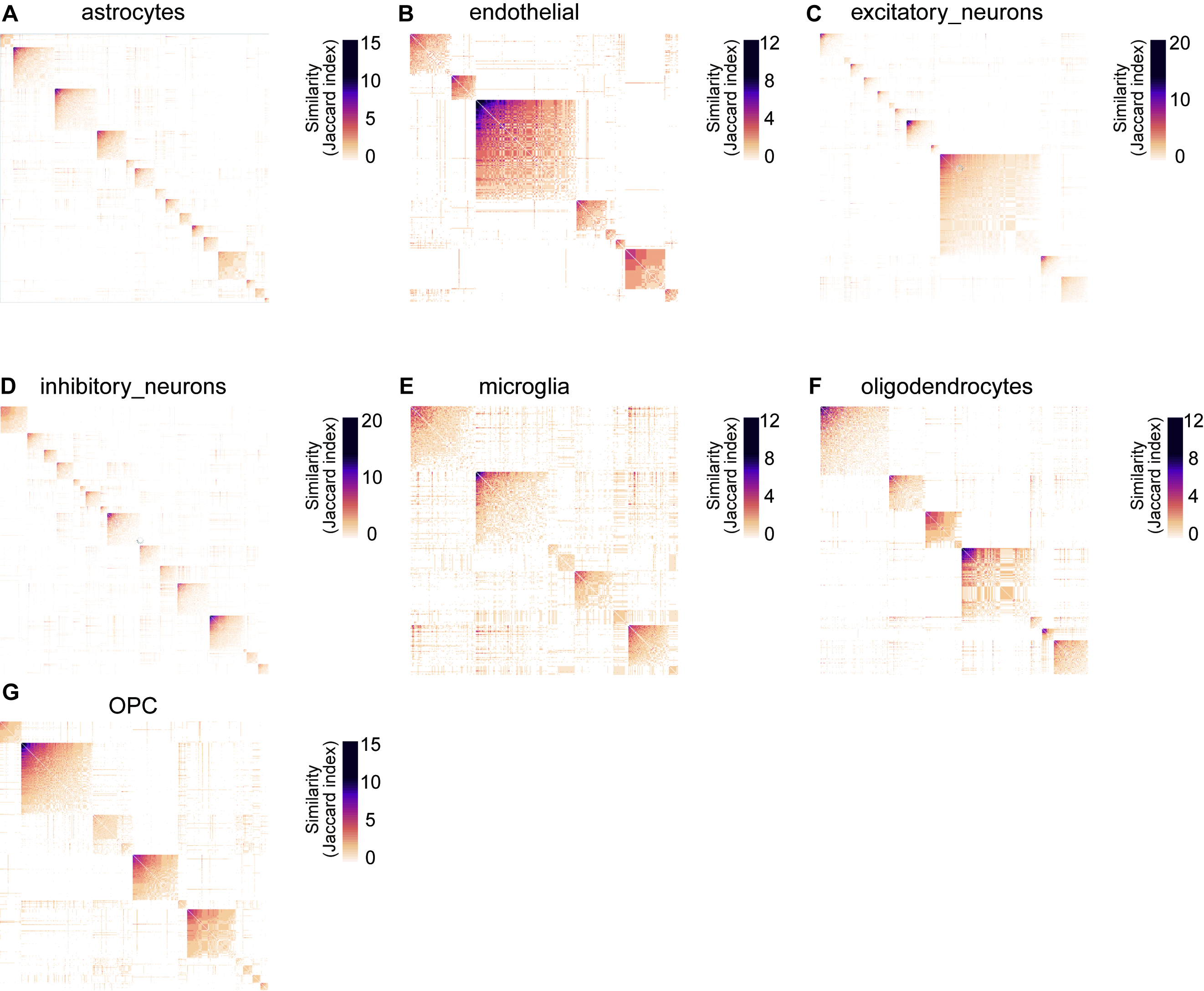

Supplement: Supplementary_material_bbag411 [file supplementary_material_bbag411.zip › Figure_S2_bbag411.tif]

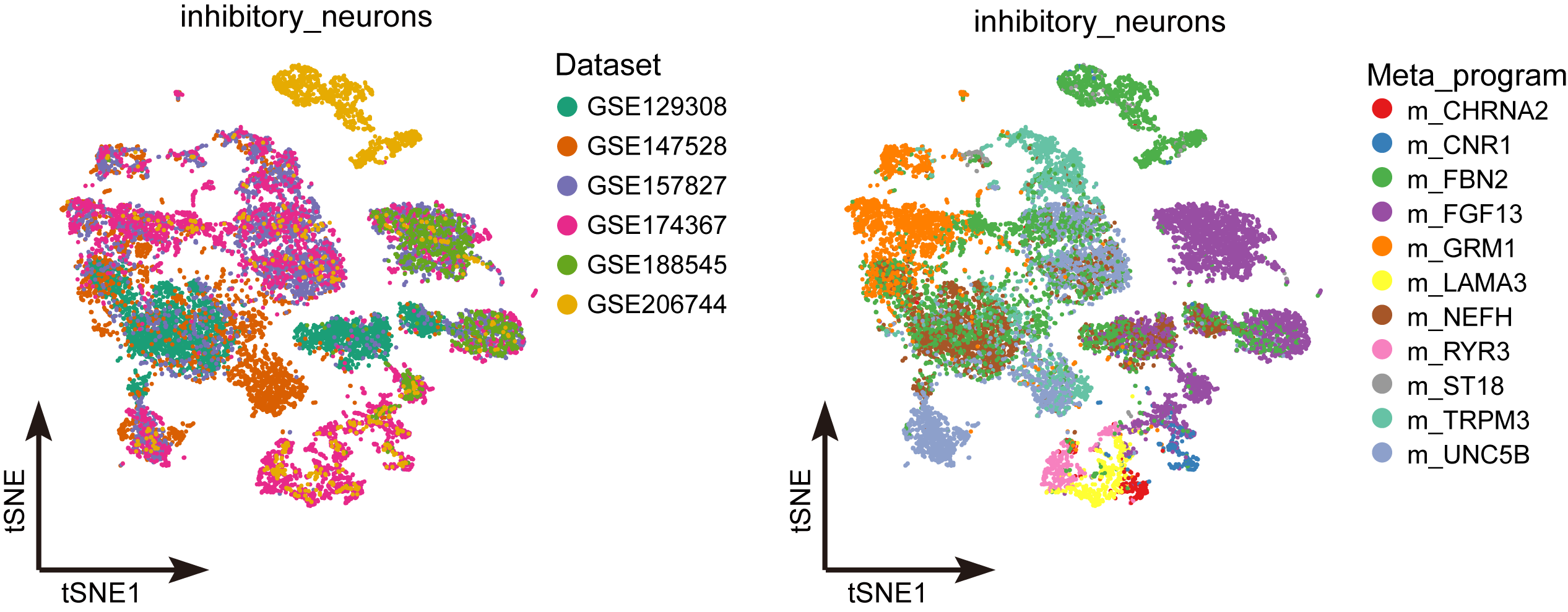

Supplement: Supplementary_material_bbag411 [file supplementary_material_bbag411.zip › Figure_S3_bbag411.tif]

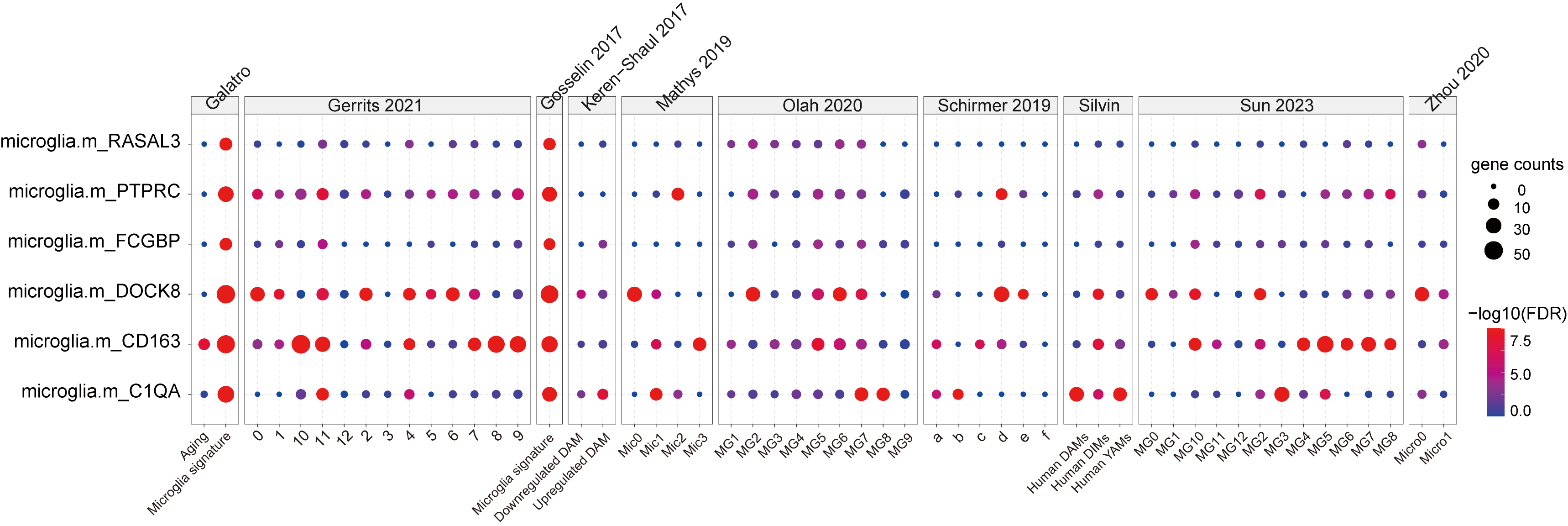

Supplement: Supplementary_material_bbag411 [file supplementary_material_bbag411.zip › Figure_S4_bbag411.tif]

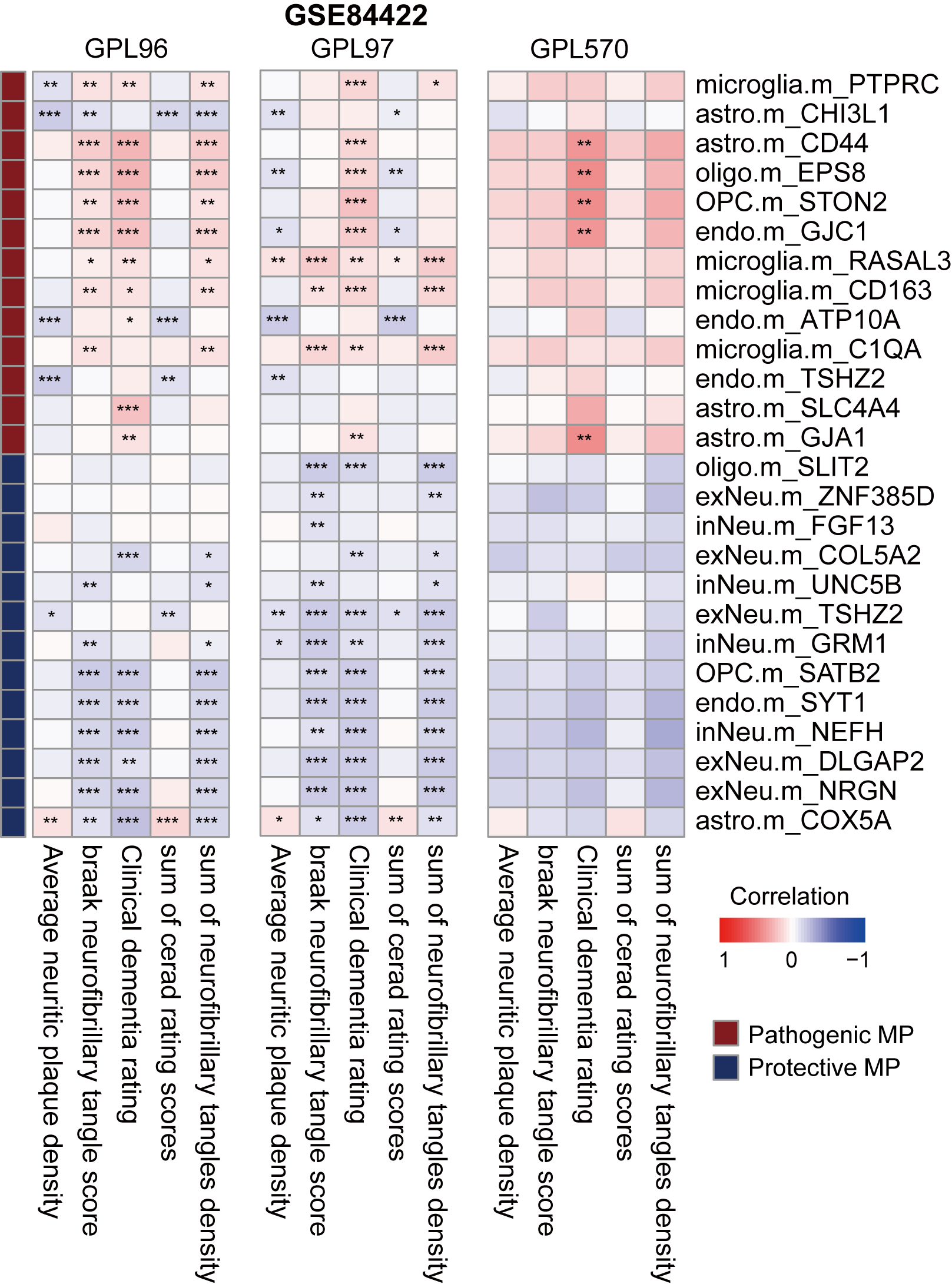

Supplement: Supplementary_material_bbag411 [file supplementary_material_bbag411.zip › Figure_S5_bbag411.tif]
